# Patient’s safety and satisfaction on same day discharge after robotic and laparoscopic radical prostatectomy versus discharge after 24 or 48 h: a longitudinal randomized prospective study

**DOI:** 10.1186/s12894-023-01318-2

**Published:** 2023-09-21

**Authors:** Eliney Ferreira Faria, Roberto Dias Machado, Rodrigo José Costa Gualberto, Marina Assunção Valadares Milani, Lucas Tadeu Bidinotto, Marcos Tobias Machado, Ricardo dos Reis, Daniele Natália Pacharone Bertolini Bidinotto

**Affiliations:** 1 Faculdade Ciências Médicas de Minas Gerais, Belo Horizonte, Brazil; 2 Department of Urology, Barretos Cancer Hospital, Rua Antenor Duarte Villela, 1331, Barretos, S. Paulo CEP 14784 400 Brazil; 3 Department of Anesthesiology, Barretos Cancer Hospital, Barretos, Brazil; 4 Molecular Oncology Research Center, Barretos Cancer Hospital, Barretos, Brazil; 5 School of Medicine, Department of Pathology, UNESP – Univ. Estadual Paulista, Botucatu, São Paulo, Brazil; 6 Barretos School of Health Sciences, Dr. Paulo Prata – FACISB, Barretos, Brazil; 7 Instituto de Cancer Arnaldo Vieira de Carvalho, Sao Paulo, Brazil; 8 Department of Gynecology, Barretos Cancer Hospital, Barretos, Brazil

**Keywords:** Early hospital discharge, Minimally invasive radical prostatectomy, Patient satisfaction, Prostatectomy

## Abstract

**Background:**

There is a tendency of prompted global health systems to reduce the length of hospital stay without compromising patient safety or satisfaction. We evaluated the safety and viability of early discharge in patients undergoing minimally invasive radical prostatectomy (MIRP), as well as patient satisfaction with this strategy.

**Methods:**

This longitudinal prospective study included 72 patients who underwent MIRP for prostate cancer. Three groups were performed according to the day of hospital discharge following surgery: same day (G1), first day after (G2), and second day after (G3). Satisfaction, adverse events, and readmission were analyzed for each group. Associations between clinicopathologic variables and same-day discharge were analyzed by comparing data between G1 patients who did and did not achieve same-day discharge.

**Results:**

16.7% of patients were not discharged according to randomization (10 randomized to G1). 80% of G1 patients who did not achieve same-day discharge had Gleason scores of 3 + 4 or 4 + 3, which were observed in 35.7% of patients discharged on the same day (P < 0.05). Average prostate weight was significantly lower in patients who achieved same-day discharge than in those who did not (P < 0.01). Univariable logistic regression points to Gleason scores of 3 + 4 or 4 + 3 as the main factors associated with unsuccessful same-day discharge (P < 0.05). There were no significant differences in satisfaction scores.

**Conclusions:**

Same-day discharge was both safe and feasible and does not appear to affect satisfaction in a subset of patients with prostate cancer. Surgeons should consider the Gleason score when determining whether same-day discharge is appropriate.

## Background

Additionally to recent advances in surgical techniques, which have substantially decreased mortality and complication rates, there is a tendency of global health systems and hospitals to reduce length of hospital stay without compromising patient safety or satisfaction [1, 2]. Minimally invasive techniques such as pure laparoscopic radical prostatectomy and robotic-assisted laparoscopic radical prostatectomy have been associated with reduced pain and intraoperative bleeding, progressively enabling early discharge for many patients [3]. This trend has been observed following several fewer complex surgeries and may soon become routine for select patients undergoing minimally invasive radical prostatectomy (MIRP), even laparoscopic or robotic assisted. Kirsh et al. [4] demonstrated the feasibility and safety of just one day of hospitalization after open radical prostatectomy in 2000, obtaining favorable results such as minimal postoperative morbidity and high patient satisfaction. A 1997 study concerning open prostatectomy also reported that reducing the duration of hospitalization had no negative effects on patient satisfaction with surgical procedures [5]. In fact, early discharge may reduce the risk of infection and allow patients to resume their general activities more quickly, promoting adherence to rehabilitation and treatment. Early discharge can also increase bed availability in hospitals and lower the cost of treatment per patient [6, 7].

While several studies have demonstrated that a high percentage of patients are eligible for discharge on the day of surgery [6, 8–10], other studies have reported failure [11, 12] or postoperative complications [13] following early discharge, highlighting the need to delineate more precise eligibility criteria. The present study aimed to investigate the safety and viability of early discharge in patients undergoing laparoscopic or robotic-assisted radical prostatectomy, meaning MIRP, as well as important variables that must be considered when determining whether patients can be discharged on the same day. Additionally, we aimed to evaluate patient satisfaction with discharge on the day of, first day after, and second day after surgery.

## Methods

This longitudinal prospective study was conducted between March 2017 and November 2019. The study was performed in accordance with the principles of the Declaration of Helsinki and approved by the Research Ethics Committee of our institution (1325/2017). Informed consent was obtained from all the participants included in the study.

The sample size was calculated using significance level of 2.5%, statistical power of 90% and margin of non-inferiority of 10 and standard deviation of 10.7, based on prospective study of Martin et al. [2]. Therefore, this study included 72 patients aged < 75 years undergoing transperitoneal laparoscopic or robotic-assisted radical prostatectomy for the primary treatment of localized prostate cancer. Additional inclusion criteria were body mass index (BMI) ≤ 35 kg/m^2^, total prostatic-specific antigen (PSA) ≤ 30 ng/ml, Gleason score ≤ 7, Briganti’s nomogram < 5%, with no requirement for lymphadenectomy [14], preoperative hemoglobin ≥ 12 g/dl, American Society of Anesthesiologists (ASA) score of I or II, and absence of cognitive impairment. Oncological and sociodemographic data were collected for all patients, who were randomized into the following three groups using REDCap software [15]: hospital discharge on the day of surgery (G1), on the first day after surgery (G2), and on the second day after surgery (G3 – standard length of hospitalization in our service, control group). There were used intermittent pneumatic compression devices and early mobilization for thromboprophylaxis in all patients. After surgery, all patients from G1 were referred to a nursing facility, where they received healthcare from the local nursing team if they need it. In order to follow up the patients discharged on the same day of surgery, they returned to the clinic for evaluation on the first day after surgery. Patients from G2 and G3 were discharged on the first and second days after surgery, respectively.

All MIRP procedures were performed at the authors’ institution starting at 7 am by the same expert surgeon (E.F.F., more than 300 cases of RARP and 500 cases of LRP). The patients received 1 g of cefazolin. Intermittent compression stockings were used during surgery, but no thromboprophylaxis was administered. The study was conducted using some recommendations from Enhanced Recovery After Surgery (ERAS) protocol [16, 17]. Foley urethral catheters and pelvic drains were used for monitoring during the post-operative period. The use of narcotics was minimized during and after the operation, and pain control was achieved mainly using nonsteroidal analgesics.

The discharge criteria included absence of fever, hemodynamic stability, oxygen saturation > 90% in atmospheric air, urine flow volume > 30 mL/h, controlled pain, tolerance to an oral diet without nausea/vomiting, and drain volume < 100 mL, removed before discharge. All patients were discharged with no drain. The exclusion criteria were blood transfusion, surgical time > 240 min, and major intraoperative complications. Patients were provided with the contact information for the team and were instructed to visit the institution’s emergency room if necessary. Patients completed the SATIS-BR questionnaire, which has been validated in Portuguese [18], as well as multiple choice questions related to their satisfaction with the length of stay and the explanation of the discharge process, among others.

Data for each group were collected at discharge and for up to 1 month after surgery. The patients returned to the hospital 8–10 days postoperatively for removal of the Foley catheter. The questionnaires were administered by a third person who was blinded to the surgical/anesthetic team and the results of randomization.

Patients were divided into the three groups at a ratio of 1:1:1. When discharge according to randomization was not possible, the patient was reallocated to a different group (defined as “migration”). Analyses among groups were performed using Fisher’s exact test or the chi-square test for categorical data and ANOVA or the Kruskal–Wallis tests for continuous data. Uni- and multivariable logistic regression was performed using the variables with P < 0.1. Statistical significance was determined based on a chi-square significance level of < 0.05. Data analysis was performed using SPSS v21 (IBM®) software.

## Results

**Fig. 1:**
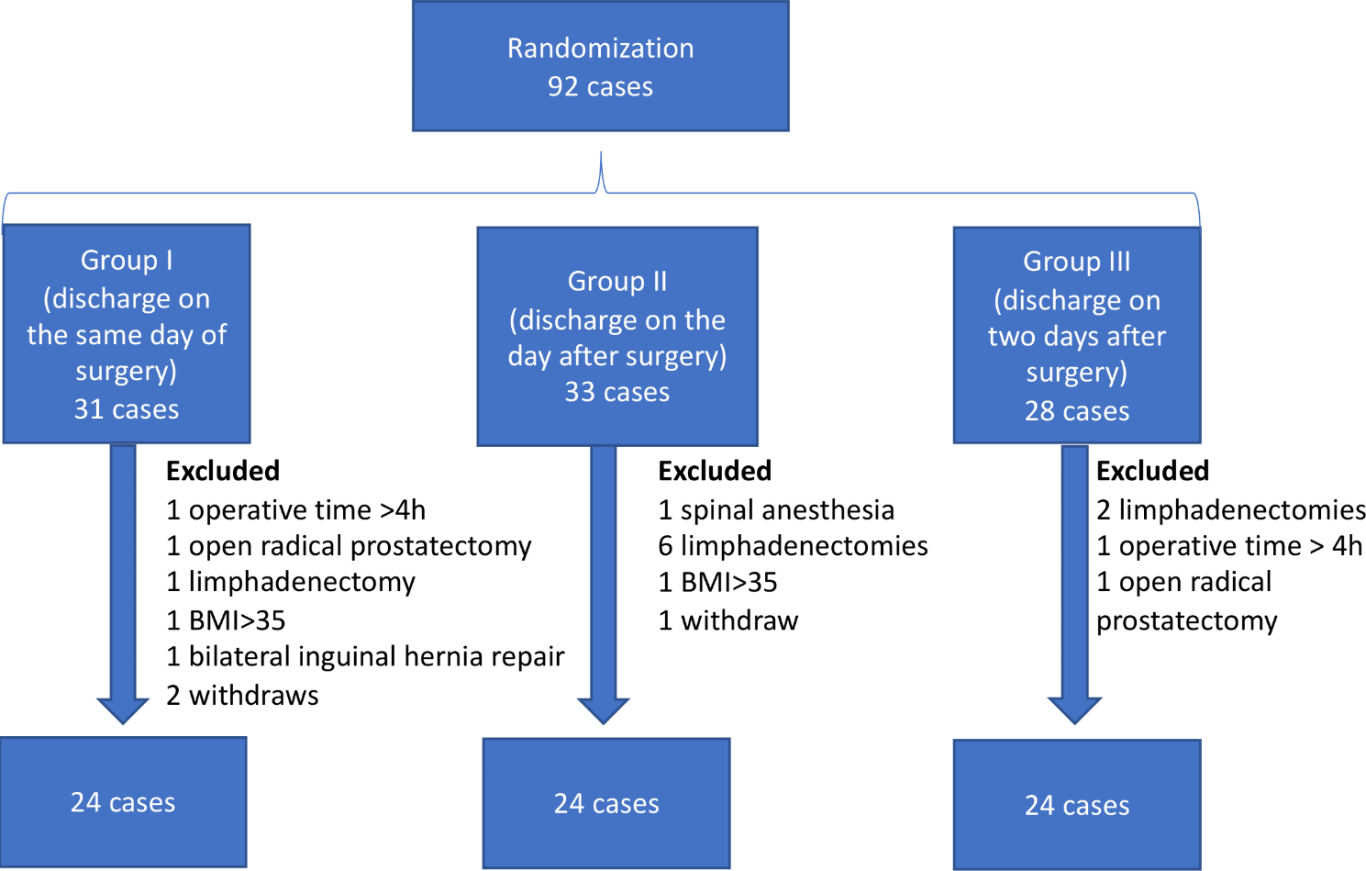
Patients recruiting design

The study included 72 patients, who randomized into three groups of 24 patients each (Fig. 1). There were no significant differences in epidemiological variables among the groups (Table 1).

**Table 1:** Epidemiological variables according to discharge group

		Discharge group			P Value
		Same day	24 h	48 h	
		N (%)			
Tobacco use	No	19 (79)	20 (83)	19 (79)	< 1
	Yes	5 (21)	4 (17)	5 (21)	
Alcohol consumption	No	13 (54)	15 (63)	15 (63)	< 1
	Yes	11 (46)	9 (38)	9 (38)	
Systemic arterial hypertension	No	14 (58)	17 (71)	17 (71)	< 1
	Yes	10 (42)	7 (29)	7 (29)	
Diabetes	No	22 (92)	23 (96)	23 (96)	< 1
	Yes	2 (8.3)	1 (4.2)	1 (4.2)	
Prior abdominal operation	No	20 (83)	17 (71)	20 (83)	< 1
	Yes	4 (17)	7 (29)	4 (17)	
TNM	T1N0M0	12 (50)	15 (63)	13 (54)	< 1
	T2N0M0	12 (25)	9 (38)	7 (46)	
Gleason score	3 + 3	11 (46)	10 (42)	13 (54)	< 1
	3 + 4	11 (46)	13 (54)	8 (33)	
	4 + 3	2 (8.3)	1 (4.2)	3 (13)	
Education level	None	3 (13)	0	2 (8.3)	< 1
	Elementary school	10 (42)	11 (46)	14 (58)	
	High school	6 (25)	7 (29)	5 (21)	
	College or higher	5 (21)	8 (25)	3 (13)	
ASA classification	I	7 (29)	3 (13)	9 (38)	< 0.3
	II	17 (71)	21 (88)	15 (63)	
		Average (standard deviation)			
Body mass index (kg/m^2^)		27.3 (2.7)	26.7 (3.5)	27.4 (4.2)	< 1
PSA (ng/mL)		5.2 (2.7)	7.5 (4.3)	7.0 (4.3)	< 0.13
Prostate weight (g)		40.2 (15.5)	36.3 (12.5)	36.1 (17.7)	< 1
Surgical time (min)		175 (0.30)	191 (0.37)	178 (0.37)	< 0.3
Blood loss (mL)		241.0 (133.9)	333.8 (178.4)	330.4 (192.2)	< 0.13

ASA: American Society of Anesthesiologists; PSA: prostate-specific antigen

Twelve patients were not discharged according to randomization, 10 of whom were previously randomized to G1 (G2 and G3, one patient each). Failure to achieve discharge on the day of surgery was related to insecurity and pain in seven patients, nausea/vomiting in two patients, and hematuria in one patient. For the two patients originally randomized to G2 and G3, the presence of mild bleeding through the drain contributed to extension of the hospital stay.

To analyze the potential associations of clinicopathologic and surgical variables with success in early discharge, we compared data between G1 patients discharged on the designated day (group 1 – G1) and those requiring an extended stay (group 1 migration – G1m). Of the variables analyzed, a Gleason score of 3 + 3 and lower prostate volume were associated with successful same-day discharge (Table 2). Univariable logistic regression of these variables shows that patients with Gleason score of 3 + 3 have 86% higher chance of receiving a successful same-day discharge than patients with score 3 + 4 or 4 + 3 (odds ratio 0.14, 95% confidence interval 0.02–0.93, P < 0.05, Table 2). Prostate volume, on the other hand, had no significative difference in univariable logistic regression. Finally, both variables had no statistical difference in multivariable statistical analysis (Table 3).

**Table 2:** Clinicopathologic and surgical data for patients randomized to same-day discharge

		Discharge group*				Univariable logistic regression		
		Same day	Same day - migration	P value	Odds ratio		95%CI**	P value
		N (%)						
Clinical stage	T1cN0M0	7 (50)	5 (50)	< 1				
	T2N0M0	7 (50)	5 (50)					
Gleason score	3 + 3	9 (64)	2 (20)	< 0.05	Ref		Ref	Ref
	3 + 4 or 4 + 3	5 (36)	8 (80)		0.14		0.02–0.93	< 0.05
Hematuria	No	7 (50)	5 (50)	< 1				
	Mild	7 (50)	4 (40)					
	Moderate	0	1 (10)					
		Average (standard deviation)						
Body mass index (kg/m^2^)		27.7 (3.2)	26.8 (3.7)	< 1				
PSA (ng/mL)***		6.6 (4.4)	5.4 (2.0)	< 1				
Prostate weight (g)		35.9 (15)	43.00 (9.9)	< 0.01	0.94		0.87–1.01	< 0.13
Surgery time		2h56 (0h36)	3h11 (0h27)	< 1				
Blood loss		294.5 (144.4)	303.2 (286.1)	< 0.3				

*Same day: patients who were discharged on the day of surgery; Same day – migration: patients who failed to be discharged on the day of surgery. **95%CI: 95% confidence interval. ***PSA: prostate-specific antigen

**Table 3:** Association between prostate volume and Gleason score (multivariable logistic regression)

			Odds ratio	95%CI*	P value
Prostate volume			0.93	0.85–1.01	0.09
Gleason score		3 + 3	ref	ref	0.05
		3 + 4 or 4 + 3	0.12	0.01–1.01	

*95%CI: 95% confidence interval

Satisfaction was analyzed both according to original group and migration group. There were no significant differences in the subscale scores (P > 0.05, Table 4). An analysis of survey responses indicated that 88% of patients considered the duration of hospitalization appropriate, while 63% of patients stated that they would not have wanted to stay in the hospital any longer. Furthermore, 89% of patients reported that they would choose the same type of surgery and duration of hospitalization again. Overall, 86% of patients reported being very satisfied, 14% reported being satisfied, and none reported being unsatisfied (Table 5).

**Table 4:** General SATIS-BR and subscale scores according to discharge group

	Discharge group*				P value
	Same day	24 h	48 h	Migration	
General**	4.9 (0.08)	5 (0.08)	4.9 (0.17)	4.9 (0.21)	< 0.13
Subscale 1**	4.9 (0.14)	5 (0.14)	4.9 (0.14)	4.9 (0.32)	< 0.13
Subscale 2**	5 (0)	5 (0)	5 (0.08)	5 (0)	< 0.13
Subscale 3**	5 (0)	5 (0)	5 (0)	4.9 (0.13)	< 1

*Migration: Patients who failed to be discharged according to the randomization group; **Values are represented as median (interquartile range).

**Table 5:** Participant responses related to the perioperative period in each subgroup

		Discharge group*				P value
		Same day	24h	48h	Migration	
		N (%)				
Do you think that your length of stay in relation to surgery was:	Very long	0	0	0	0	< 1
	Long	1 (7.1)	0	1 (4.3)	0	
	Adequate	11 (79)	21 (92)	21 (92)	10 (83)	
	Short	2 (14)	2 (8.7)	1 (4.3)	2 (17)	
	Very short	0	0	0	0	
Would you like to have been in the hospital longer?	Certainly yes	0	0	0	0)	< 1
	I think so	1 (7.1)	2 (8.7)	2 (8.7)	1 (8.3)	
	I am not sure	0	3 (13)	0	0	
	I do not think so	4 (29)	2 (8.7)	7 (30)	5(42)	
	Certainly not	9 (64)	16 (70)	14 (61)	6 (50)	
Are you satisfied with the type of anesthesia used in your surgery?	Very unsatisfied	0	0	0	0	< 1
	Unsatistied	0	0	0	0	
	I am not sure	0	0	0	0	
	Satisfied	2 (21)	4 (17)	7 (30)	3 (25)	
	Very satisfied	11 (79)	19 (83)	16 (70)	9 (75)	
After surgery, how did you feel with the pain medication prescribed?	Pain all the time	0	0	0	1 (8.3)	< 0.13
	Pain most of the time	1 (7.1)	0	0	0	
	I’m not sure	0	1 (4.3)	0	0	
	No pain most of the time	6 (43)	14 (61)	10 (44)	9 (75)	
	No pain at all	7 (50)	8 (35)	13 (57)	2 (17)	
Did you feel satisfied with the explanations of the discharge process?	Very unsatisfied	0	0	0	0	< 1
	Unsatisfied	1 (7.1)	0	0	0	
	I am not sure	0	2 (8.7)	0	0	
	Satisfied	6 (43)	8 (35)	7 (30)	6(50)	
	Very satisfied	7 (50)	13 (57)	16 (70)	6(50)	
If you could go back in time again, would you choose the way your surgery and hospitalization were performed?	Certainly not	1 (7.1)	0	1 (4.3)	0	< 1
	I do not think so	0	0	0	0	
	I am not sure	2 (14)	1 (4.3)	1 (4.3)	0	
	I think so	1 (7.1)	0	1 (4.3)	0	
	Certainly yes	10 (71)	22 (96)	20 (87)	12 (100)	
In general, did you stay:	Very unsatisfied	0	0	1 (4.3)	0	< 1
	Unsatisfied	0	0	0	0	
	I am not sure	0	0	0	0	
	Satisfied	2 (14)	3 (13)	4 (17)	3 (25)	
	Very satisfied	12 (86)	20 (87)	18 (78)	9 (75)	

*Migration: Patients who failed to be discharged according to the randomization group

No adverse events related to patient safety were observed in any of the three groups. In addition, no significant differences were observed between patients who remained in the discharge group and those who were not discharged according to randomization. There were no cases of readmission.

## Discussion

MIRP has several advantages over traditional procedures, including reduced blood loss and transfusion rates, decreased postoperative pain, and a shorter duration of hospitalization in more recent cases [19]. The current findings suggest that discharge on the day of surgery is both safe and feasible for a subset of patients undergoing MIRP. Our analysis also indicated that a Gleason score of 3 + 3 is the main factor that significantly increases the success of discharge on the day of surgery. Notably, all patients in our study were satisfied with the procedure and the length of stay.

Historically, the duration of hospitalization following open RP has decreased with increasing surgical experience over the last 100 years, as noted by Klein et al. [1]. In their study, reducing the median length of stay from 7 to 2 days following open radical prostatectomy (RP) did not significantly change mortality or new hospitalization/complication rates, although nearly 90% of patients reported overall satisfaction. A few years later, Martin et al. [2] were the first to report the results of a feasibility study involving 11 patients undergoing outpatient open retropubic RP. Pure laparoscopic and robot-assisted RP have also been associated with decreases in postoperative length of stay, pain, and blood loss when compared with traditional open procedures [3]. Dudderidge et al. reported that 78% of patients who underwent conventional laparoscopic RP were discharged after one night of hospitalization, while 7% of patients were discharged on the same day [20].

Several high-volume surgical centers have described their initial experience regarding the safety and viability of outpatient robot-assisted laparoscopic radical prostatectomy (RARP), reporting similar complication and outcome rates [6, 12, 21, 22]. Research has further indicated that RARP increases patient satisfaction and reduces postoperative pain levels when compared with open procedures. In addition to reducing recovery time, these improvements allow patients to resume their general activities more quickly and enhance their perception of their own general health [23]. Another important factor would be the type of surgery. It is well known that the preservation of peritoneal cavity integrity may ensure an earlier recovery of intestinal activity, a more rapid return to diet and, consequently, a shorter length of stay. However, the protocol of our center is the transperitoneal route [24], and still did not impact negatively our results.

Berger et al. [12] conducted a prospective study involving 30 patients, 87% of whom were discharged on the day of surgery. In their study, they observed no significant differences in demographic or perioperative variables between the outpatient and hospitalization groups. In our study, 12 patients were not discharged according to randomization due to mild pain, nausea/vomiting, or hematuria and related insecurities. These results indicate that, even when early discharge is considered clinically safe, patients must have the option to stay if they do not feel safe. In the study by Berger et al. [12], four patients were not discharged on the day of surgery, three of whom elected to forego early discharge despite having no clinical problems.

These data reinforce the notion that patient selection and motivation are important factors influencing same-day discharge from the hospital following surgery. Similarly, the support network provided at hospital discharge is also important success in the immediate postoperative period. The use of an intermediate care hospital, established in a municipality, reduced length of stay without increasing readmissions, admissions, mortality, activities of daily living, primary health care utilization or total care days [25]. Ensuring initial assistance in a support home monitored by a technical nursing assistant may help to improve acceptance and success of early discharge. However, it is well known that support homes and/or nursing facilities are not present worldwide. Therefore, convenient access to the medical team via electronic communication, and early outpatient follow up may decrease postoperative anxiety among postsurgical patients. In our study, we had no post-surgical complications nor readmission, showing that these selected patients were safe with an early discharge.

Khalil et al. [10] analyzed postoperative data for patients undergoing RARP using the database of the American College of Surgeons National Surgical Quality Improvement Program. The data were used to identify patients who had been discharged from the hospital on the day of surgery (n = 258) and those who had stayed in the hospital for more than 1 day (n = 1,290). Global morbidity, reoperation, and readmission rates were low and did not significantly differ between the two groups. Abboudi et al. [13] analyzed data for 32 patients who underwent laparoscopic RP and were discharged on the day of surgery. Postoperative complications were observed in six patients, including intensive care unit (ICU) admission, lymphocele infection, and re-catheterization secondary to a defective catheter balloon. Hospitalization was necessary in four of these six cases. Berger et al. [12] reported no differences in perioperative or functional outcomes between individuals undergoing outpatient RARP and a compatible inpatient group. Banapour et al. [21] reviewed data for 51 patients who underwent RARP, 51% of whom underwent an ambulatory procedure. No differences in operative time, blood loss, or complication rates were observed between patients discharged early and those requiring a standard hospital stay. Several other centers have reported their experience with early discharge, noting that same-day discharge following RARP does not appear to increase postoperative complication or readmission rates when compared with a standard overnight stay [6, 10, 12, 21]. Together, these studies have provided no clear evidence that reducing the duration of hospitalization leads to increases in complication rates following RP. Our data support this notion, as we observed excellent short-term results without cases of readmission or decreases in patient satisfaction.

Despite promising evidence, relevant studies have included well-selected patient populations with limited comorbidities, an ideal BMI, and adequate social support. Most patients included in the study of outpatient RARP by Khalil et al. [10] were young, were not current smokers, had a low ASA class, and did not have obesity. The authors also reported a shorter operative time and a reduced need for pelvic lymphadenectomy in patients undergoing RARP. Our study included patients without obesity and those with localized disease not requiring pelvic lymphadenectomy. Notably, researchers have highlighted the relationship of obesity and comorbidities with an increased risk of complications and prolonged recovery time after RARP [26]. The present study found, in these well-selected patients, that Gleason score was the main variable that should be considered during the decision of discharging patient in the same day of surgery. Besides another Brazilian study [11], that found prostate volume as a factor, our univariable logistic regression pointed to Gleason score as more important, and prostate volume was a potential confusing variable. It is well known that prostate volume can impact in perioperatory results, such as blood loss and surgery time [27–30]. In fact, higher Gleason scores increase the chance to surgical margins involvement, extracapsular invasion and/or seminal vesicle involvement, leading to the need of a more complex surgery and/or involving bigger surgical margins [31, 32].

In addition to the feasibility and safety of early discharge of this selected population, we found that satisfaction of the patients was high, independently on discharge time. This result corroborates several reports [2, 5, 13, 33] that applied satisfaction questionnaires to patients (11, 129, 32 and 100 patients, respectively) after early discharge, and found that satisfaction was uniformly high.

As surgeons have become more experienced with RARP, some of the initially restrictive criteria used for patient selection, such as BMI or the need for lymph node dissection, have been expanded in more recent series [6, 10]. Khalil et al. [10] reported that > 70% of outpatient surgery cases occurred after 2012. All single-center studies on laparoscopic RP or outpatient RARP were published after 2010 [2, 10, 12, 21]. This may be related to the learning curve, confidence in the methodology, and standardization of MIRP, which has progressed with increasingly lower blood transfusion and complication rates [34, 35]. In fact, Ploussard et al. [36] performed a countrywide study of the RARPs performed in France in 2020 and found association of same-day discharge and higher-volume centers, which gives the notion that, besides very well solid criteria of patient selection, the experience of surgeon is fundamental for early day discharge success. In the current study, all surgical procedures were performed by surgeons experienced in minimally invasive surgery at high-volume oncological centers.

This study had several strengths when compared with previous investigations. To the best of our knowledge, this is the first prospective, randomized study of early discharge after RP conducted at one of the largest cancer hospitals in Latin America. Our findings expand the body of knowledge regarding safety and patient satisfaction in cases of same-day discharge after surgery and provide insight into factors that may predict success following early discharge.

Despite these strengths, our study also had some limitations. Although the calculation of the number of patients was performed under very well delimited parameters, the sample size is not extensive. It is well known that patient satisfaction surveys can suggest positive results even when the results are poor. To limit the influence of such bias, we utilized a validated satisfaction instrument (SATIS-BR), which was administered shortly after discharge by a third researcher blinded to the patient groups. Furthermore, given that procedures were performed by surgical experts at high-volume centers, no patients in our study experienced perioperative complications, which naturally increased the likelihood of high satisfaction scores regardless of hospitalization time. Additionally, some questions possibly were not well-understood by the patients, which could have biased some results. For example, one patient of the same-day discharge group answered that felt that the length of his stay was long. Lastly, while the study was conducted at a public hospital, participants exhibited significant differences in socioeconomic status.

## Conclusions

The findings of this study indicate that same-day discharge is both safe and feasible following RARP and does not decrease patient satisfaction rates. For discharge on the day of surgery to be feasible at a large scale, routine surgical changes are required to minimize the risk of adverse events [37]. In this study, we utilized some recommendations from ERAS protocol [38], which has been shown to reduce the duration of hospitalization and treatment cost without influencing complication or readmission rates [17, 39]. Furthermore, surgeons performing RARP should carefully select and motivate patients when making decisions regarding same-day discharge, which require consideration of the Gleason score.

## Data Availability

The datasets used and analysed during the current study are available from the corresponding author on reasonable request.

## List of abbreviations

ASA: American Society of Anesthesiologists
BMI: body mass index
ERAS: Enhanced Recovery After Surgery
ICU: intensive care unit
MIRP: minimally invasive radical prostatectomy
PSA: prostatic-specific antigen
RARP: robot-assisted laparoscopic radical prostatectomy
REDCap: Research Electronic Data Capture
RP: radical prostatectomy
